# The role of the microbiome and psychosocial stress in the expression and activity of drug metabolizing enzymes in mice

**DOI:** 10.1038/s41598-020-65595-9

**Published:** 2020-05-22

**Authors:** Nina Zemanová, Pavel Anzenbacher, Iveta Zapletalová, Lenka Jourová, Petra Hermanová, Tomáš Hudcovic, Hana Kozáková, Martin Vodička, Jiří Pácha, Eva Anzenbacherová

**Affiliations:** 1 Department of Medical Chemistry and Biochemistry, Faculty of Medicine and Dentistry, Palacky University Olomouc, Olomouc, Czech Republic; 2 Department of Pharmacology, Faculty of Medicine and Dentistry, Palacký University, Olomouc, Czech Republic; 3 Institute of Microbiology, Academy of Sciences of the Czech Republic, Nový Hrádek, Czech Republic; 4 Institute of Physiology, Academy of Sciences of the Czech Republic, Prague, Czech Republic

**Keywords:** Enzymes, Metabolism

## Abstract

The gut microbiota is involved in a number of different metabolic processes of the host organism, including the metabolism of xenobiotics. In our study, we focused on liver cytochromes P450 (CYPs), which can metabolize a wide range of exo- and endogenous molecules. We studied changes in mRNA expression and CYP enzyme activities, as well as the mRNA expression of transcription factors that have an important role in CYP expression, all in stressed germ-free (GF) and stressed specific-pathogen-free (SPF) mice. Besides the presence of the gut microbiota, we looked at the difference between acute and chronic stress. Our results show that stress has an impact on CYP mRNA expression, but it is mainly chronic stress that has a significant effect on enzyme activities along with the gut microbiome. In acutely stressed mice, we observed significant changes at the mRNA level, however, the corresponding enzyme activities were not influenced. Our study suggests an important role of the gut microbiota along with chronic psychosocial stress in the expression and activity of CYPs, which can potentially lead to less effective drug metabolism and, as a result, a harmful impact on the organism.

## Introduction

The gut microbiota is a community of microorganisms consisting not only of bacteria, but also of archaea, viruses and unicellular eukaryotes^1^. This extended microbial genome (microbiome) contains more than 5 million unique genes and has a significant impact on the host organism^2^. The microbiome is involved in a number of different metabolic processes, from physiological ones, such as the digestion of complex polysaccharides into short-chain fatty acids, development of the immune system, metabolism of fatty acids, biosynthesis of neurotransmitters^3,4^, to pathophysiological processes (during dysbiosis) such as obesity, inflammatory bowel disease, diabetes, colorectal cancer and allergies^5^. In addition, studies have shown that the gut microbiota has a substantial role in the bioavailability and metabolism of xenobiotics^6^. Biotransformation enzymes involved in the metabolism of exogenous and endogenous compounds can be affected directly or indirectly by the gut microbiota. Directly, by their various metabolic reactions, such as reductive and hydrolytic reactions, decarboxylation, dealkylation, dehalogenation and deamination. Indirectly, by affecting the modulation of host drug and xenobiotic metabolism^7^.

In this study, we focused on drug-metabolizing cytochromes P450 (CYPs) - an enzyme superfamily of haem-containing monooxygenases. CYPs can metabolize a wide range of structurally diverse exogenous molecules. However, they are also able to catalyze many specific reactions of endogenous compounds, such as the biosynthesis of steroid hormones, prostaglandins or bile acids^8,9^. Over 50 000 known CYP enzymes are divided into families and subfamilies based on their sequence similarity, but only several enzymes belonging to the CYP 1, 2 and 3 families participate in drug and xenobiotic metabolism^10^.

The expression and function of the majority of CYPs is multifactorially controlled by genetic polymorphisms, epigenetic influences and non-genetic factors such as sex, age, hormonal influence and diseases^9^. Therefore, knowledge of all the factors influencing the function of CYPs is essential for the prediction of pharmacokinetics and drug response.

CYP gene expression is controlled by transcription factors such as aryl hydrocarbon receptor (AhR), constitutive androstane receptor (CAR), pregnane X receptor (PXR) and peroxisome proliferator-activated receptor alpha (PPARα)^9^. AhR was firstly recognized because of its role in modulating the response to 2,3,7,8-tetrachlorodibenzo-p-dioxin (TCDD) and it regulates the transcription of CYP1A1, CYP1A2 and CYP1B1^11^. CAR was first linked to the phenobarbital-mediated induction of the CYP2B gene subfamily (e.g. CYP2B6). It also controls the expression of CYP3A4, CYP2A6 and CYP2Cs^12^. PXR was named based on its activation by endogenous C21 steroids (pregnanes)^13^. According to many studies, PXR is considered the mediator of CYP3A regulation^14^. PPARα is mainly involved in lipid and energy homeostasis. However, PPARα can directly regulate CYP3A4 transcription and is involved in the constitutive and inducible regulation of CYP2C8^15^.

Another factor affecting CYP regulation, which has now been studied intensively, is inflammation. The suppression of CYPs mediated by pro-inflammatory mediators has been systematically shown in human hepatocyte cultures. On the other hand, the alterations of the selected mediators does not represent the complexity of the organism and the overall effect of multiple cytokines (pro- and anti-inflammatory) in acute and chronic inflammation^16^.

Inflammation and immune changes have also been associated with stress after a large number of studies over the years^17^. The term “stress” describes the effects of any threats that could disrupt homeostasis^18^. Physiological response to stressors is mediated by the hypothalamic-pituitary–adrenal (HPA) axis and the central and peripheral autonomic nervous system. The final effectors of the stress response systems are glucocorticoids. Response to stress also involves the neuroendocrine, cellular and molecular system^19^. At the molecular level, stress (both acute and chronic) has been shown to be able to change parameters of the immune system^20^.

We assume that psychosocial acute and chronic stress (associated with inflammation and immune changes) influences CYP-dependent xenobiotic metabolism as well as the respective transcription factors. In this work, we tried to contribute to the discussion on the role of the gut microbiome by investigating the effect of the presence of the gut microbiome and psychosocial stress on the regulation of the main enzymes of drug biotransformation, cytochromes P450.

## Results

### mRNA expression of transcription factors (chronic vs. acute stress)

**Figure 1:**
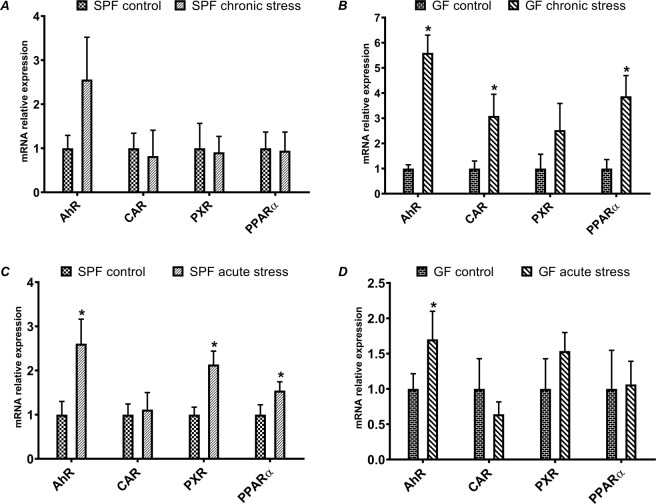
Relative mRNA expression of transcription factors in chronically stressed mice (**A** – SPF mice, **B** – GF mice) and in acutely stressed mice (**C** – SPF mice, **D** – GF mice). (*Significantly different from control (p ≤ 0,05)).

First, we focused on the mRNA expression of transcription factors in the liver tissue of the specific-pathogen-free (SPF) mice, either chronically or acutely stressed (Fig. 1A, resp. 1 C). In parallel, we investigated the same patterns of expression in germ-free (GF) mice, i.e. chronically and acutely stressed mice without the presence of the microbiome (Fig. 1B, resp. 1D). The amount of mRNA is expressed as a relative expression.

The following transcription factors were chosen - aryl hydrocarbon receptor (AhR), constitutive androstane receptor (CAR), pregnane X receptor (PXR) and peroxisome proliferator-activated receptor alpha (PPARα). The mRNA expression of the transcription factors (AhR, CAR and PPARα) was significantly affected (upregulated) in GF chronically stressed mice (Fig. 1B) compared to SPF mice exposed to the same, i.e. chronic stress (Fig. 1A). Moreover, the relative mRNA expression of AhR in GF chronically stressed mice was more than 6 times higher than in GF control mice (Fig. 1B). These results show that the mRNA regulation of transcription factors in GF mice is much more sensitive to chronic stress than in SPF mice.

The mRNA expression of the transcription factors in acutely stressed mice is different in GF and SPF mice (Fig. 1D, resp. 1 C), when compared to chronically stressed mice. In GF mice, only the AhR expression was significantly increased by acute stress. On the other hand, in SPF acutely stressed mice, we observed increased mRNAs of AhR, PXR and PPARα.

### mRNA expression and enzyme activity of CYPs (chronic vs. acute stress)

In the following real-time qPCR experiment, we concentrated on the mRNA expression of CYPs from families 1, 2 and 3, namely CYP1A1, 1A2, 2A5/4, 2B10, 2C29, 2C38, 2D22, 2E1, 3A11 and 3A13. Here we focused on the CYP mRNA expression, again in SPF and GF mice, when exposed to chronic or acute stress. The amount of mRNA is expressed as relative expression.

**Figure 2:**
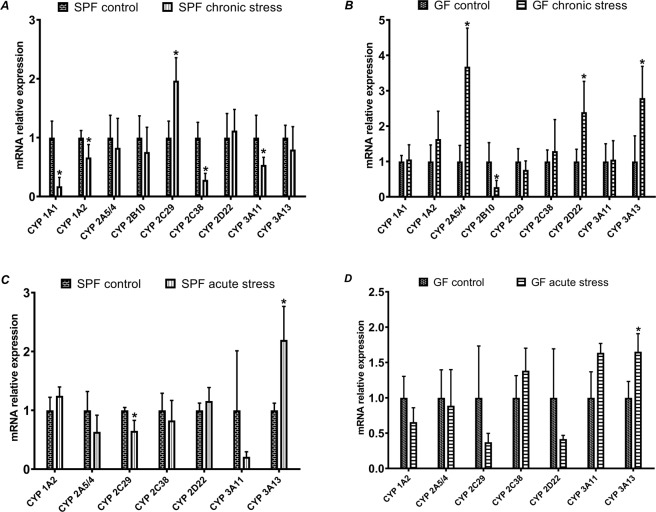
Relative mRNA expression of cytochromes P450 in chronically stressed mice (**A** – SPF mice, **B** – GF mice) and in acutely stressed mice (**C** – SPF mice, **D** – GF mice). (*Significantly different from control (p ≤ 0,05)).

Significant changes were found in chronically stressed mice, either SPF or GF. Interestingly, as with the expression of transcription factors, there were differences between SPF and GF mice exposed to chronic stress. In the GF animals, we observed an increase in the mRNA level for CYP2A5/4, 2D22 and 3A13, whereas the mRNA expression of CYP2B10 was significantly decreased (Fig. 2B). However, we observed a different response in SPF chronically stressed mice, with most of the mRNA expression levels (CYP1A1, 1A2, 2C38 and 3A11) being lower than in SPF control mice (Fig. 2A). The only expression that was higher than the control was CYP2C29.

On the contrary, compared with chronically stressed mice, fewer significant changes were observed in the CYP mRNA expression of acutely stressed mice. The GF acutely stressed mice were not affected as much as GF chronically stressed mice. The only significantly affected CYPs were CYP2C29 in SPF acutely stressed mice (a decreased level of mRNA) and CYP3A13, which was increased in both GF and SPF acutely stressed mice (Fig. 2C,D). We also observed that there is a higher variability in GF acutely stressed mice than in SPF mice.

**Figure 3:**
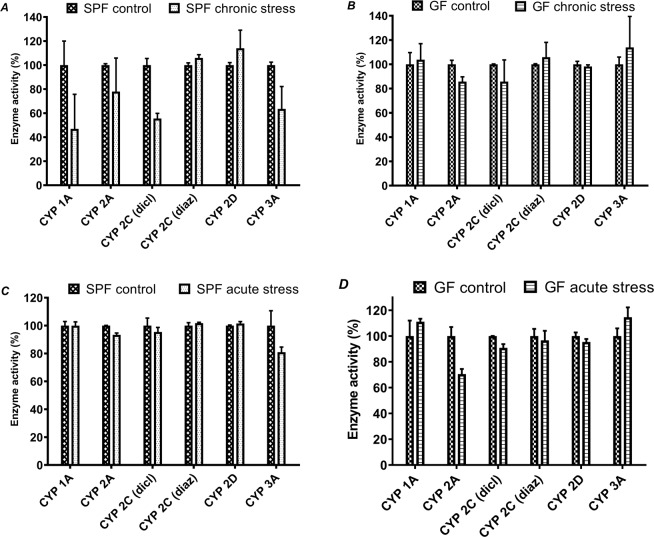
Enzyme activity of cytochromes P450 in chronically stressed mice (**A** – SPF mice, **B** – GF mice) and in acutely stressed mice (**C** – SPF mice, **D** – GF mice).

The enzyme activities of CYPs did not exhibit significant differences in response to acute stress in the SPF and GF animals (Fig. 3C,D). In SPF chronically stressed mice (Fig. 3A), the enzyme activity of CYP2C (determined with diclofenac as substrate) and CYP3A was decreased – CYP2C by 44,4% and CYP3A by 36,4% in comparison to control SPF mice. In contrast, acute stress did not significantly affect the activities (Fig. 3C,D).

### Comparison of effect of acute and chronic stress - mRNA expression of transcription factors and CYPs in SPF and GF mice

**Figure 4:**
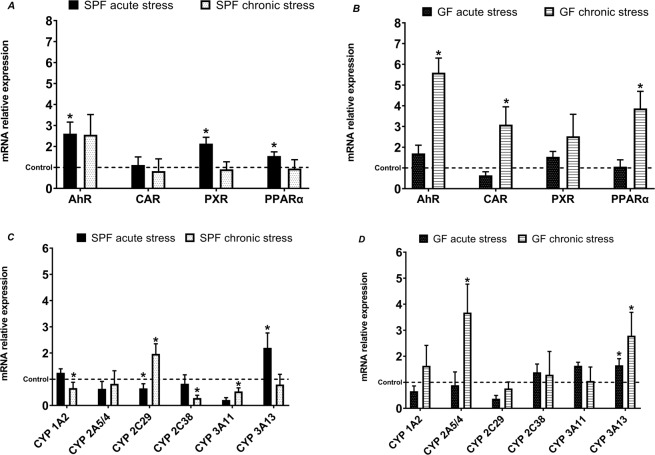
Comparison of effect of acute and chronic stress in mRNA expression of transcription factors in SPF (**A**) and GF (**B**) mice, and in mRNA expression of CYPs in SPF (**C**) and GF (**D**) mice. (*Significantly different from control (p ≤ 0,05).

Another perspective on the obtained data was the comparison of acute vs. chronic stress. The expression of transcription factors (Fig. 4) was most affected in chronically stressed GF mice (Fig. 4B) compared to SPF mice (Fig. 4A), indicating that the GF mice were more sensitive to chronic stress. In other words, in the GF mice, the mRNA level of transcription factors such as AhR, CAR and PPARα increased with chronic stress (Fig. 4B). However, the acute stress had a bigger impact on the expression of transcription factors in SPF mice, where the mRNA levels of AhR, PXR and PPARα were increased (Fig. 4A).

The CYP expression in GF stressed mice, however, was less affected than the SPF stressed mice (Fig. 4C,D). In GF mice, the expression of CYP2A5/4 was affected by chronic stress (expression is increased) and only CYP3A13 was affected by both acute and chronic stress (increase in mRNA level) (Fig. 4D). In SPF mice (Fig. 4C), CYP expression was affected differently: chronic stress mainly caused a significant decrease in the mRNA of CYP1A2, 2C38 and 3A11, except for an increase in CYP2C29. Acute stress significantly affected CYP2C29 (decrease) and CYP3A13 (increase).

## Discussion

The expression of CYPs is influenced by many factors, including genetic polymorphisms, the presence of xenobiotics, inflammation, hormones and age^9^. The results of this study prove that stress has an important impact on the metabolism of drugs and xenobiotics in general. Stress is able to alter constitutive and induced CYPs. The adrenergic receptor-linked pathways and glucocorticoids appear to have an important role in the stress-mediated regulation of CYPs^19^. Interestingly, we can observe differences in effects between chronic and acute stress as well as differences in response between SPF and GF stressed murine models. So far, it has been proven that intestinal bacteria can alter the expression of biotransformation enzymes^21,22^. Selwyn and colleagues analyzed the hepatic transcriptome of GF and SPF mice by RNA-Seq and compared the mRNA expression of drug-metabolizing enzymes. They further proved that the absence of microbiota alters the expression of drug-metabolizing enzymes, such as CYPs^22^.

Overall, we observed an increased mRNA expression of transcription factors, both in GF and SPF mice. When comparing chronic and acute stress, we saw that even short-term stress can change expression in SPF mice. However, in GF chronically stressed mice, we observed significantly increased expressions of AhR, CAR and PPARα, which were not increased in SPF mice, so we may suppose that under chronic stress, the gut microbiome contributes to maintaining homeostasis. Interestingly, stress has a great impact on the mRNA expression of AhR. AhR is a ligand-activated factor and regulates many genes, including the CYP1A1/2, CYP1B1, TCDD - inducible poly(ADP-Ribose) polymerase (TIPARP) and aryl hydrocarbon receptor repressor (AHRR). According to recent studies, AhR can act as a modulator of immune and inflammatory responses. In *AhR -/-* mice with dextran sulfate sodium (DSS)-induced colitis, higher levels of pro-inflammatory cytokines were observed in comparison to wild-type controls, indicating that AhR plays an important role in the regulation of pro-inflammatory cytokines^23^. In our experiment, the relative expression of AhR was more than five times higher in GF chronically stressed mice, than the GF control (Fig. 1B). Interestingly, it was also significantly increased in acutely stressed mice, both GF and SPF (Fig. 1C,D). We suggest that AhR expression increases to manage stress, and that it is needed to reduce the impact of pro-inflammatory cytokines. We also observed the impact of gut microbiota. The mRNA expression level of AhR was higher in GF chronically stressed mice compared to SPF stressed mice. However, AhR is not the only transcription factor that has a role in inflammation, or in inflammatory response. Recent studies suggest that PXR and CAR can also regulate energy homeostasis and immune response^24^. We observed a significant increase in CAR (GF chronically stressed mice, Fig. 1B) and PXR (SPF acutely stressed mice, Fig. 1C).

In addition, PPARα, a ligand-activated nuclear receptor, appears to have a role in suppressing inflammation^25^. In GF chronically stressed mice, the expression of PPARα was more than 3.5 times higher than in control GF mice (Fig. 1B). According to these results, the importance of the microbiome in the adaptation to chronic stress is evident. Nevertheless, the gene expression of particular CYPs is usually regulated by more than one transcription factor and is itself a relatively complex process.

Acute stress causes changes in the nervous, cardiovascular, endocrine and immune system which are generally adaptive^18^. In acutely stressed mice, even though the expression of transcription factors (AhR, PXR and PPARα) was increased, we observed that the only significant changes were in the mRNA expression of CYP3A13 (↑ in GF and SPF mice) and CYP2C29 (↓ in SPF mice). We also observed inter-individual variation among mice, so it may be assumed that acute stress does not have a considerable impact on CYP mRNA expression. This finding, that acute stress does not significantly affect CYPs, is also supported by the enzyme activities of CYPs, as no CYP enzyme activity was significantly changed.

The difference between acute and chronic stress appears when the acute stress response becomes maladaptive after being repeatedly activated. Then the chronic stress suppresses the immune system, directly by affecting cytokines, due to elevated basal levels of stress hormones^18^. Studies have proved that inflammation can caused a downregulation of the majority of CYP enzymes^26^. The impact of the immune system, or the effect of inflammation on the CYP enzymes, has been studied extensively. One of the best-studied models of inflammation, exposure to bacterial lipopolysaccharide (LPS), caused changes in CYP expression in LPS-treated murine liver^27^. The greatest impact on CYP regulation in the liver was found to be by inflammatory cytokines, such as interleukin 1β (IL-1β), interleukin 6 (IL-6), tumor necrosis factor α (TNFα) and interferons α and γ. These cytokines can regulate different CYPs, so perhaps that is why they are not only downregulated, and the expression of CYPs can be unaffected or even upregulated^28^.

In recent years, a connecting element between chronic stress, the immune system and microbiota has been found - the microbiota-gut-brain axis. The microbiota-gut-brain axis, as the name suggests, is a bidirectional signaling pathway between the gut microbiota and the brain, and it is regulated at the neural, endocrine and immune level^29^. There is also evidence of the gut microbiota influencing stress-related behaviors, and studies have proved that modified microbiota (by an altered diet) had an impact on reducing stress-related behavior^30^.

According to our study, chronic stress along with the gut microbiota changes the mRNA expression and enzyme activity of liver CYPs. In both SPF and GF mice, increased mRNA expression is not directly reflected in enzyme activities. In GF mice, an increase in the mRNA expression of CYP2A5/4, 2D22 and 3A13 was observed, but activities were not significantly different compared to the control. On the other hand, in SPF mice, we were able to see the effect of the gut microbiota and chronic stress, as the expressions of CYP1A1, 1A2, 2C38 and 3A11 were significantly decreased. The activities of CYP2C (using diclofenac as substrate) and CYP3A were also decreased.

Interestingly, there is no direct correlation between the levels of mRNA of transcription factors and some CYPs. The expression of transcription factors is either unaffected or significantly increased, whereas the CYP expression shows not only an increase, but also a decrease in mRNA levels. We assume that under the chronic stress condition, the increase in expression of mRNA of transcription factors has its own role in inflammatory responses and it may not be simply reflected in CYP expression.

## Materials and Methods

### Animals

The animal experiment realized in this study is a version of method described in our previous work^31^. Two-month-old germ-free (GF) and specific pathogen-free (SPF) male BALB/c mice were used for experiments. GF mice were kept under sterile conditions in Trexler-type plastic isolators. One month before the beginning of the experiments, the SPF mice were transferred to similar isolators to ensure identical conditions for all groups during the experiments. Animals were kept on a 12-h light/dark cycle with unrestricted access to autoclaved tap water and a 50 kGy-irradiated sterile pellet diet of Altromin 1410 (Altromin, Lage, Germany). The GF mice were monitored weekly for the presence of aerobic and anaerobic bacteria, molds and yeast contamination by standard microbiological methodologies. SPF mice were fed the same sterile diets as GF mice. The experiments were approved by the Committee for the Protection and Use of Experimental Animals of the Institute of Microbiology, Academy of Sciences of the Czech Republic (approval ID: 78/2014). The methods were carried out in strict accordance with the approved guidelines.

### Experimental design – models of chronic and acute stress

The social defeat chronic stress used in this study was induced according to the resident-intruder paradigm used in our previous work^32^. The procedure is based on the fact that a male mouse will defend its territory against an unfamiliar male intruder. GF (n = 7) and SPF (n = 7) male mice designated as residents (older, sexually experienced males) were housed individually for 7 days before the experiment without a change of bedding (a manipulation often used to enhance territoriality and aggression). Unstressed GF (n = 7) and SPF (n = 6) (control) mice were undisturbed in their home cage; moreover, control mice never witnessed the stress procedure, because the resident-intruder interaction was never done in isolators containing unstressed animals.

Acute stress was induced according to the method of Zimprich^33^, in which GF (n = 9) and SPF (n = 14) mice were subjected to 2-hour restraint stress in plastic 50 ml tubes, equipped with ventilation holes. Unstressed (control) GF (n = 9) and SPF (n = 12) were undisturbed in their home cage.

After the final stress session, the animals were anesthetized with isoflurane vapor. Isoflurane was chosen because it does not interfere with gene transcriptional responses and the acute and chronic stress responses stay intact^34^. The anesthetized mice were decapitated and the liver was removed, weighed and kept frozen until the next procedure. Liver tissue samples used for qPCR analysis were stored in RNA later (Qiagen, Hilden, Germany).

### RNA isolation, reverse transcription and real-time quantitative polymerase chain reaction analysis

Total RNA was isolated from murine liver tissues using an RNeasy Mini Kit (Qiagen, Hilden, Germany) following the manufacturer’s protocol. The concentration of RNA in each sample was quantified spectrophotometrically at 260 nm using a NanoPhotometer® N60 (Implen, Munich, Germany).

RNA was then transcribed to single-stranded cDNA using a Transcriptor High-Fidelity cDNA Synthesis Kit (Roche, Basel, Switzerland). The expression of CYPs and transcription factors was quantified by real-time qPCR performed in a LightCycler 1536 Instrument (Roche, Basel, Switzerland) using specific TaqMan Gene Expression Assays by Thermo Fisher Scientific (Life Technologies, Prague, Czech Republic) (see Supplemental Data). Miniaturized qPCR in 1536-well format plates were pipetted using Echo Liquid Handler (Labcyte, Dublin, Ireland). Calculations were based on the 2(-Delta Delta C(T)) method^35^. The values of each target gene were normalized to the expression of the housekeeping gene - hypoxanthine guanine phosphoribosyl transferase (*Hprt*).

### Liver microsomal fractions

Murine livers were pooled into individual groups (in total six groups; GF control and GF stressed, SPF control and SPF stressed, for both acutely and chronically stressed mice). The microsomal preparation was according to the established protocol^36^ and each microsomal fraction was stored at −80 °C. Concentrations of cytochrome P450 were determined spectrophotometrically^37^, providing information on the sum of concentrations of native CYP forms in the sample.

### Cytochrome P450 enzyme activity assays

The enzyme activities of individual CYPs were measured in the murine hepatic microsomal fractions according to the established protocols. For enzyme activity assays of individual CYPs, the prototypic substrates were used: CYP1A1/2–7-ethoxyresorufin, CYP2A – coumarin, CYP2C – diazepam, CYP2C – diclofenac, CYP2D – bufuralol^38^ and CYP3A – midazolam^39^. Incubation mixtures contained potassium phosphate buffer (pH 7.4), NADPH-generating system (NADP^+^, isocitrate, isocitrate dehydrogenase and MgCl_2_), liver microsomes and the substrate. Detailed descriptions of the conditions of the individual activity assays can be found in Table 1. A Shimadzu LC-20 HPLC system (Shimadzu, Kyoto, Japan) with UV or fluorescence detection was used for the determination of metabolites. The measurements were performed in a LiChrospher RP-18 column or a Chromolith® High Resolution RP-18 endcapped column (determination of midazolam substrate) (Merck, Germany). The HPLC conditions are given in Table 1.

**Table 1:** Conditions and HPLC parameters for the measurement of enzyme activity assays.

CYP	Substrate	Metabolite	Substrate concentration(µM)	pmol of CYP/ incubation volume (µl)	Quenching agent	Elution	Injection of sample (µl)	Detection	
								UV (nm)	Fluorescence Ex/Em(nm)
1A1/2	7-Ethoxyresorufin	Resorufin	2.6	35/100	100% methanol	Isocratic	50		535/585
2 A	Coumarin	7-Hydroxycoumarin	6.25	35/100	100% methanol	Isocratic	5		338/458
2 C	Diazepam	Desmethyldiazepam	100	70/200	100% acetonitrile	Isocratic	50	229	
2 C	Diclofenac	4´-Hydroxydiclofenac	16	35/200	Acetonitrile/Acetic acid (94:6)	Binary gradient	50	280	
2D	Bufuralol	1´-Hydroxybufuralol	25	67.3/200	70% HClO_4_	Binary gradient	5		252/302
3 A	Midazolam	1´-Hydroxymidazolam	2.8	12.56/100	100% methanol	Isocratic	50	240	

### Statistical analysis

Data are expressed as means **±** SD. The statistical significance of gene expression was determined by unpaired Student’s t-test, using Statistica software version 12 (Statsoft CR, Prague, Czech Republic). When p ≤ 0.05, data were regarded as statistically significant. Due to the scarcity of material, the statistical significance of activity assays could not be determined.

## Supplementary information

Supplemental Data.

## Data Availability

All data generated or analysed during this study are included in this published article (and its Supplementary Information file).

## References

[1] D’Argenio V, Salvatore F (2015). The role of the gut microbiome in the healthy adult status. Clin Chim Acta.

[2] Walker AW, Duncan SH, Louis P, Flint HJ (2014). Phylogeny, culturing, and metagenomics of the human gut microbiota. Trends Microbiol.

[3] Lankelma JM, Nieuwdorp M, de Vos WM, Wiersinga WJ (2015). The gut microbiota in internal medicine: implications for health and disease. Neth J Med.

[4] Lynch SV, Pedersen O (2016). The Human Intestinal Microbiome in Health and Disease. N Engl J Med.

[5] Claus SP, Guillou H, Ellero-Simatos S (2016). The gut microbiota: a major player in the toxicity of environmental pollutants?. NPJ Biofilms Microbiomes.

[6] Spanogiannopoulos P, Bess EN, Carmody RN, Turnbaugh PJ (2016). The microbial pharmacists within us: a metagenomic view of xenobiotic metabolism. Nature reviews. Microbiology.

[7] Wilson ID, Nicholson JK (2017). Gut microbiome interactions with drug metabolism, efficacy, and toxicity. Transl Res.

[8] Park BK, Pirmohamed M, Kitteringham NR (1995). The role of cytochrome P450 enzymes in hepatic and extrahepatic human drug toxicity. Pharmacol Ther.

[9] Zanger UM, Schwab M (2013). Cytochrome P450 enzymes in drug metabolism: regulation of gene expression, enzyme activities, and impact of genetic variation. Pharmacol Ther.

[10] Guengerich, F. P. In Cytochrome P450: Structure, Mechanism, and Biochemistry (ed. Paul R. Ortiz de Montellano) 523-785 (Springer International Publishing (2015).

[11] Korecka A (2016). Bidirectional communication between the Aryl hydrocarbon Receptor (AhR) and the microbiome tunes host metabolism. NPJ Biofilms Microbiomes.

[12] Yang H, Wang H (2014). Signaling control of the constitutive androstane receptor (CAR). Protein Cell.

[13] Xing Y, Yan J, Niu Y (2020). PXR: a center of transcriptional regulation in cancer. Acta Pharm Sin B.

[14] Timsit YE, Negishi M (2007). CAR and PXR: the xenobiotic-sensing receptors. Steroids.

[15] Thomas M (2015). Peroxisome proliferator-activated receptor alpha, PPARalpha, directly regulates transcription of cytochrome P450 CYP2C8. Front Pharmacol.

[16] Harvey RD, Morgan ET (2014). Cancer, inflammation, and therapy: effects on cytochrome p450-mediated drug metabolism and implications for novel immunotherapeutic agents. Clinical pharmacology and therapeutics.

[17] Slavich GM, Irwin MR (2014). From stress to inflammation and major depressive disorder: a social signal transduction theory of depression. Psychol Bull.

[18] Schneiderman N, Ironson G, Siegel SD (2005). Stress and health: psychological, behavioral, and biological determinants. Annu Rev Clin Psychol.

[19] Konstandi M, Johnson EO, Lang MA (2014). Consequences of psychophysiological stress on cytochrome P450-catalyzed drug metabolism. Neurosci Biobehav Rev.

[20] Segerstrom SC, Miller GE (2004). Psychological stress and the human immune system: A meta-analytic study of 30 years of inquiry. Psychological Bulletin.

[21] Bjorkholm B (2009). Intestinal microbiota regulate xenobiotic metabolism in the liver. PloS one.

[22] Selwyn FP, Cui JY, Klaassen CD (2015). RNA-Seq Quantification of Hepatic Drug Processing Genes in Germ-Free Mice. Drug metabolism and disposition: the biological fate of chemicals.

[23] Neavin, D. R., Liu, D., Ray, B. & Weinshilboum, R. M. The Role of the Aryl Hydrocarbon Receptor (AHR) in Immune and Inflammatory Diseases. *Int J Mol Sci* 19, 10.3390/ijms19123851 (2018). 10.3390/ijms19123851 PMC6321643 30513921

[24] Gao J, Xie W (2012). Targeting xenobiotic receptors PXR and CAR for metabolic diseases. Trends Pharmacol Sci.

[25] Kaipainen A (2007). PPARalpha deficiency in inflammatory cells suppresses tumor growth. PloS one.

[26] Zidek Z, Anzenbacher P, Kmonickova E (2009). Current status and challenges of cytokine pharmacology. Br J Pharmacol.

[27] Richardson TA, Morgan ET (2005). Hepatic cytochrome P450 gene regulation during endotoxin-induced inflammation in nuclear receptor knockout mice. J Pharmacol Exp Ther.

[28] Aitken AE, Richardson TA, Morgan ET (2006). Regulation of drug-metabolizing enzymes and transporters in inflammation. Annu Rev Pharmacol Toxicol.

[29] Kelly JR (2015). Breaking down the barriers: the gut microbiome, intestinal permeability and stress-related psychiatric disorders. Front Cell Neurosci.

[30] Foster JA, Rinaman L, Cryan JF (2017). Stress & the gut-brain axis: Regulation by the microbiome. Neurobiol Stress.

[31] Kozakova H (2016). Colonization of germ-free mice with a mixture of three lactobacillus strains enhances the integrity of gut mucosa and ameliorates allergic sensitization. Cellular & molecular immunology.

[32] Vodicka M (2018). Microbiota affects the expression of genes involved in HPA axis regulation and local metabolism of glucocorticoids in chronic psychosocial stress. Brain Behav Immun.

[33] Zimprich A (2014). A robust and reliable non-invasive test for stress responsivity in mice. Front Behav Neurosci.

[34] Wu XY (2015). Effect of pentobarbital and isoflurane on acute stress response in rat. Physiol Behav.

[35] Livak KJ, Schmittgen TD (2001). Analysis of relative gene expression data using real-time quantitative PCR and the 2(-Delta Delta C(T)) Method. Methods.

[36] Lake, B. G. in *Biochemical Toxicology*, *A Practical Approach* Ch. 183-215, (IRL Press (1987).

[37] Schenkman JB, Jansson I (1998). Spectral analyses of cytochromes P450. Methods Mol Biol.

[38] Phillips I, Shephard E (2006). Cytochrome P450 Protocols. Humana Press.

[39] Kronbach T, Mathys D, Umeno M, Gonzalez FJ, Meyer UA (1989). Oxidation of Midazolam and Triazolam by Human-Liver Cytochrome P450iiia4. Molecular Pharmacology.

