# Supplementary material for: The role of the microbiome and psychosocial stress in the expression and activity of drug metabolizing enzymes in mice
Source: Sci Rep. 2020 May 22;10:8529. doi: 10.1038/s41598-020-65595-9 (PMC7244717; doi:10.1038/s41598-020-65595-9)
Supplement: Supplementary file 1 — Supplemental Data. [file 41598_2020_65595_MOESM1_ESM.docx]

**The role of the microbiome and psychosocial stress in the expression and activity of drug metabolizing enzymes in mice**

Nina Zemanová, Pavel Anzenbacher, Iveta Zapletalová^,^ Lenka Jourová, Petra Hermanová, Tomáš Hudcovic, Hana Kozáková, Martin Vodička, Jiří Pácha and Eva Anzenbacherová

**Supplemental Data**

List of primers used for qPCR.

Commercial TaqMan Gene Expression Assays were ordered from Thermo Fisher Scientific, Life Technologies, Prague, Czech Republic.

| Gene Symbol | Assay ID |
| --- | --- |
| Cyp1a1 | Mm00487218_m1 |
| Cyp1a2 | Mm00487224_m1 |
| Cyp2a5/4 | Mm00487248_g1 |
| Cyp2b10 | Mm00456591_m1 |
| Cyp2c29 | Mm00725580_s1 |
| Cyp2c38 | Mm00658527_m1 |
| Cyp2d22 | Mm00530542_m1 |
| Cyp2e1 | Mm00491127_m1 |
| Cyp3a11 | Mm00731567_m1 |
| Cyp3a13 | Mm00484110_m1 |
| Hprt | Mm03024075_m1 |
| Ahr | Mm00478932_m1 |
| Nr1i3 | Mm01283978_m1 |
| Nr1i2 | Mm01344139_m1 |
| Ppara | Mm00440939_m1 |
